# REST missense mutations reveal disrupted Re1 motif binding and co-repressor interactions in uterine fibroids

**DOI:** 10.3389/fbinf.2025.1703356

**Published:** 2026-01-12

**Authors:** Srineevas Sriram, Chandresh Palanichamy, P. T. Subash, Manshi Kumari Gupta, C. Sudandiradoss

**Affiliations:** Department of Biotechnology, School of Biosciences and Technology, Vellore Institute of Technology, Vellore, Tamil Nadu, India

**Keywords:** MD simulations, protein-protein docking, protein-DNA docking, re1 motif, REST (re1-silencing transcription factor), uterine fibroids

## Abstract

**Introduction:**

The Re1-Silencing Transcription Factor (REST) is a master regulator of gene silencing, orchestrating transcriptional repression by tethering chromatin-modifying co-repressors to the Re1 motif of target genes. While REST is recognized as a sentinel of cellular identity, its role in uterine fibroids (UF) remains unclear. This study aims to investigate how structural perturbations in REST may compromise its regulatory function and contribute to altered transcriptional control in fibroid biology.

**Methods:**

A deep structural interrogation of REST was performed through expansive *in silico* analysis of 938 missense SNPs. Evolutionary conservation was assessed across ten primate species to identify structurally disruptive variants. Structural modelling, protein–protein and protein–DNA docking analyses were conducted to evaluate interactions with co-repressors and DNA. Molecular dynamics simulations were used to assess conformational stability, flexibility, compactness, and energetic changes in wild-type and mutant REST variants.

**Results:**

Five structurally disruptive REST variants (Y31C, Y31D, L76Q, Y283C, L427Q) were identified at evolutionarily conserved residues. Structural modelling and docking analyses revealed weakened affinity for co-repressors, with the Y283C variant showing a marked reduction in SIN3A interaction (Z-score: 2.4 to −1.2) and impaired DNA binding (Z-score: 2.0 to −1.3). Molecular dynamics simulations demonstrated that Y283C increased rigidity (RMSF: 0.33 to 0.27 nm), reduced compactness (Rg: 3.48–3.51 nm), and lowered potential energy. Upon Re1 binding, destabilization intensified, with increased RMSD (0.95–1.07 nm) and pronounced shifts in energy.

**Discussion:**

This integrative analysis highlights REST as a candidate regulatory component in uterine fibroid biology. Structural disruption of REST, particularly through the Y283C mutation, may destabilize molecular interactions and compromise DNA-binding precision, potentially unleashing transcriptional noise that fuels fibroid growth. These findings suggest that perturbation of REST-mediated transcriptional repression may be associated with altered regulatory control in this disease and could inform future strategies to investigate dysregulation in uterine fibroids.

## Graphical Abstract

**Figure:**
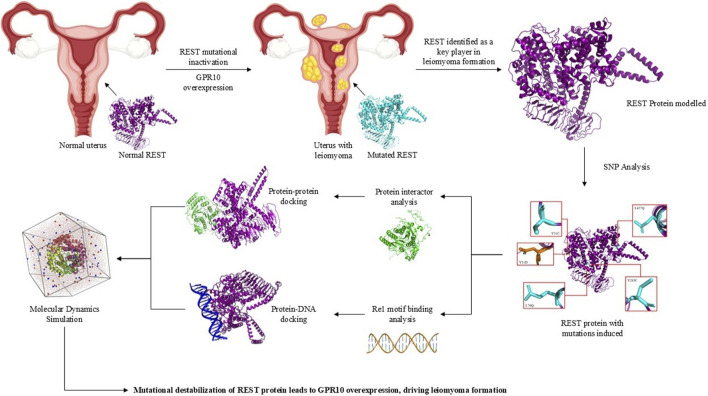
Investigating the Role of REST Mutational Destabilization in Uterine Leiomyoma Formation via In Silico Docking and Molecular Dynamics Simulations. This graphical abstract depicts the computational workflow exploring the impact of single nucleotide polymorphisms (SNPs) on REST protein stability and its role in uterine leiomyoma formation. Homology modelling of REST was followed by SNP-based mutational induction, with destabilizing mutations predicted through stability analysis. Protein-protein and protein-DNA docking were employed to assess the effects of mutations on REST interactions with key partners and the Re1 motif. Molecular dynamics (MD) simulations were then performed to evaluate the structural destabilization of REST mutants, linking mutational inactivation to GPR10 overexpression, a proposed driver of uterine leiomyoma pathogenesis.

## 1 Introduction

Uterine leiomyomas (uterine fibroids; UF) are the most common benign pelvic tumors affecting women of reproductive age, posing a significant clinical and public health challenge (Baird et al., 2015). They arise from smooth muscle cells of the myometrium and are characterized by excessive extracellular matrix deposition. UF affects an estimated 70%–75% of women and contributes substantially to gynecological morbidity, reduced quality of life, and major healthcare costs, with annual economic burden in the United States estimated between $5.9 and $34.3 billion (Mauskopf et al., 2005; Bulun, 2013). Despite their benign nature, the widespread occurrence and debilitating symptoms of UF necessitate continued research into their molecular underpinnings, pathophysiology, and potential therapeutic interventions.

Beyond their high prevalence, uterine fibroids are frequently associated with other gynecological conditions, including endometriosis, adenomyosis, infertility, ovarian dysfunction, and, in rare cases, malignant transformation into leiomyosarcoma (Devaud et al., 2022). The overlapping symptomatology and pathogenic mechanisms between these disorders complicate diagnosis and treatment. Endometriosis and adenomyosis share common hallmarks with fibroids, such as hormonal dysregulation, chronic inflammation, and aberrant extracellular matrix remodeling, which can amplify disease severity. Fibroid-induced infertility arises from mechanical disruption of the uterine environment, impairing implantation and pregnancy maintenance. In cases of ovarian dysfunction, fibroids contribute to menstrual irregularities and hormonal imbalances, further exacerbating reproductive challenges. The complexity of fibroid pathophysiology underscores the need for targeted, non-invasive therapeutic approaches (Gupta et al., 2008).

Despite their prevalence, the molecular mechanisms underlying UF pathogenesis remain not fully elucidated, limiting the development of effective targeted therapies. Emerging evidence implicates aberrant gene expression patterns and dysregulated signaling pathways in UF formation, with recent studies suggesting the involvement of transcriptional repressors in tumorigenesis. Among these, REST, a key regulator of neuronal gene expression, has gained attention for its potential role in non-neuronal tissues, including the reproductive system (Rueda and Davis, 2013).

Recent studies have highlighted the critical role of the PI3K/AKT/mTOR signaling pathway in the pathogenesis of uterine fibroids, linking dysregulated upstream signaling molecules to fibroid growth (Karra et al., 2009). The overexpression of GPR10, also known as the prolactin-releasing hormone receptor (PRLHR), has been identified as the most highly upregulated G protein-coupled receptor (GPCR) in human fibroid tissues. Although the primary role of GPR10 is associated with prolactin regulation in the hypothalamus, its ubiquitous overexpression in fibroid cells has been shown to drive cell proliferation via PrRP-mediated activation (Gu et al., 2004; Samson and Taylor, 2006). Notably, REST has been demonstrated to transcriptionally repress GPR10 in cell lines (Cloud et al., 2022; Varghese et al., 2013), further supporting the link between REST inactivation, GPR10 overexpression, and PI3K/AKT/mTOR pathway activation in uterine fibroids. The absence of functional REST results in aberrant GPR10 expression, which promotes fibroid cell proliferation and contributes to the pathogenesis of leiomyomas (Rueda and Davis, 2013).

**FIGURE 1:**
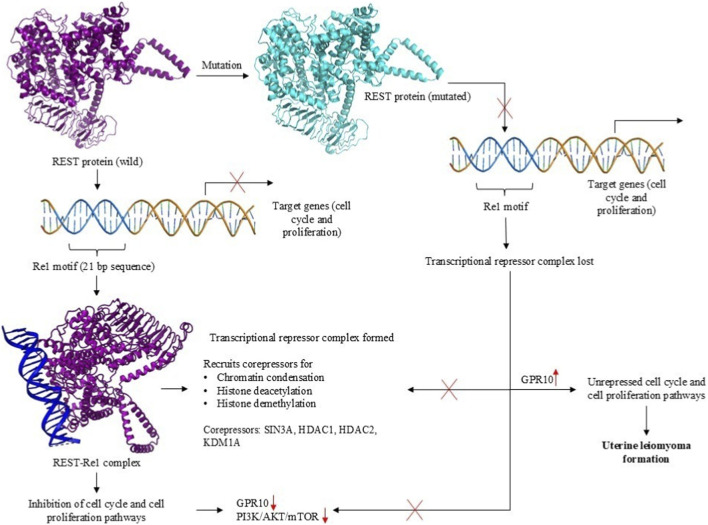
Mechanistic Insights into REST Mutational Destabilization Driving Uterine Leiomyoma Formation via GPR10 Overexpression. This schematic represents the proposed molecular mechanism by which single nucleotide polymorphisms (SNPs) in the REST gene contribute to uterine leiomyoma formation. In the wild-type state, REST binds to the Re1 motif on target gene promoters, recruiting corepressors such as SIN3A, HDAC1, HDAC2, and KDM1A to form a transcriptional repressor complex. This complex inhibits cell cycle and proliferation pathways, including GPR10-mediated PI3K/AKT/mTOR signaling. However, mutational destabilization of REST impairs its DNA-binding ability, leading to the loss of transcriptional repression, GPR10 overexpression, and consequent activation of cell cycle and proliferation pathways, driving uterine leiomyoma pathogenesis.

Given REST’s role in transcriptional repression and its involvement in fibroid pathogenesis through GPR10 dysregulation, it is crucial to explore the mechanisms by which REST interacts with its corepressor complexes to regulate gene expression. REST exerts its tumor-suppressive function by repressing the expression of genes involved in cell cycle progression and proliferation. It binds to specific DNA sequences known as Re1 motifs (Otto et al., 2007) and recruits corepressor complexes, including SIN3A, HDAC1, HDAC2, KDM1A, and RBBP7 (Xiao et al., 2021) forming multiprotein complexes like SIN3A-HDAC (Huang et al., 1999) and CoREST-KDM1A (Ismail et al., 2018; Kalin et al., 2018; Kim et al., 2020) among others. These complexes modulate chromatin structure through histone deacetylation (Tiana et al., 2017), histone demethylation (Kim et al., 2020), and chromatin condensation, creating a repressive environment that silences target genes. This repression inhibits cell cycle regulators and prevents excessive cell proliferation (Leighton and Williams, 2019). However, mutations or dysregulation of REST disrupts the recruitment of these corepressors, leading to the loss of transcriptional repression. This results in the overexpression of GPR10, which activates the PI3K/AKT/mTOR signaling pathway. The involvement of REST in these interconnected pathways highlights its crucial role in maintaining cellular homeostasis and its potential as a therapeutic target in fibroid treatment (Figure 1).

Understanding REST’s role in uterine fibroids offers promising insights into their pathogenesis. By repressing genes linked to cell proliferation through chromatin remodeling, REST helps maintain cellular balance. Its dysregulation may promote fibroid growth via GPR10 and PI3K/AKT/mTOR pathways. Targeting REST and its interactors could pave the way for innovative, non-invasive therapies for Uterine Fibroids.

## 2 Materials and methods

### 2.1 Dataset collection

The idea to perform a computational analysis of the REST protein stemmed from the studies conducted by Rueda and Davis, (2013), and Cloud et al. (2022). The study by Rueda and Davis focused on the theoretical understanding of the REST protein in Uterine Leiomyomas, and the study by Cloud et al. was an experimental study that proved that REST dysregulation resulted in Uterine leiomyomas by characterisation studies conducted on REST cKO mice under a myometrial-specific iCre (MiC) ablation.

The amino acid sequence for REST protein was retrieved from the Universal Protein Database (Bateman et al., 2022). This sequence was used as an input in *in silico* tools to generate three-dimensional structures of REST protein as well as to predict the deleteriousness of its SNPs. The missense SNPs were collated into a list from the dbSNP database of NCBI (Sherry et al., 2001), which was then used in the SNP analysis.

### 2.2 Generating three-dimensional structure of REST

The 3D structure for REST protein was generated using the AlphaFold3 program via the Chai-1 web server on Neurosnap.ai (Abramson et al., 2024; Boitreaud et al., 2024), a state-of-the-art deep learning-based protein structure prediction tool. The primary amino acid sequence of REST (UniProt ID: Q13127) was submitted to the AlphaFold3 platform, yielding a high-confidence structural model with domain-wise per-residue confidence scores.

Validation of the predicted structure was performed by plotting their pLDDT values, extracted from the B-factor column of their PDB files, against the residue numbers. The resulting 3D model served as the basis for downstream docking and molecular dynamics (MD) simulations as well as structure-based functional analyses.

The structure was further validated using ERRAT score (Colovos and Yeates, 1993), a measure of the quality of a protein structure, and PROCHECK (Laskowski et al., 1993), to determine the Ramachandran contact criteria and stereochemical properties of the protein residues by generating a Ramachandran plot, both of which were available on the SAVES v6.1 web server made by UCLA-DOE LAB. The best model was then refined using GalaxyRefine (Ko et al., 2012) and re-evaluated to ensure that it was ready for simulation.

### 2.3 Evaluation of deleteriousness of REST SNPs

Five *in silico* tools (SIFT (Ng and Henikoff, 2001), PolyPhen-2 (Adzhubei et al., 2013), PhD-SNPg (Capriotti and Fariselli, 2017), mCSM (Pires et al., 2013), DynaMut2 (Rodrigues et al., 2020)) were used to assess mutation deleteriousness based on sequence homology, conservation, and structural data. The final five missense variants were prioritized based on consensus predictions across multiple computational tools, representing those with the strongest predicted structural and functional impact. Because the focus of this study was on protein destabilization rather than population frequency or clinical association, variant selection was intentionally based on computational consensus rather than epidemiological filtering. Following the SNP analysis, a consensus approach was used to shortlist the top five most deleterious SNPs which were consistently predicted to be damaging across all the tools.

### 2.4 Multiple sequence alignment of REST

To investigate the evolutionary conservation of functionally significant residues in REST, a multiple sequence alignment (MSA) was performed using its protein sequences from ten primate species: *Homo sapiens* (human), *Pan paniscus* (bonobo), *Pan troglodytes* (chimpanzee), *Nomascus leucogenys* (northern white-cheeked gibbon), *Hylobates moloch* (silvery gibbon), *Pongo pygmaeus* (Bornean orangutan), *Chlorocebus sabaeus* (green monkey), *Macaca mulatta* (rhesus macaque), *Macaca fascicularis* (crab-eating macaque), and *Macaca nemestrina* (southern pig-tailed macaque). The purpose of this analysis was to assess the evolutionary significance of key REST residues associated with transcriptional repression and to determine whether specific missense mutations in REST could have functional consequences in the context of uterine fibroids.

REST protein sequences were initially aligned using the Basic Local Alignment Search Tool for Proteins (BLAST-P) (Camacho et al., 2009) to confirm their similarity and ensure accurate selection of homologous regions. Subsequently, Clustal Omega (Sievers and Higgins, 2013) was employed to generate the final MSA. Default parameters were used for alignment, with the inclusion of gap penalties and sequence weighting to optimize accuracy. The conservation of critical REST residues, particularly those affected by missense mutations (Y31C, Y31D, L76Q, Y283C, and L427Q), was assessed across species.

### 2.5 Network analysis

To compile a comprehensive list of REST interactors, a multidimensional approach was employed, combining an extensive literature review with network analysis tools. The initial step involved reviewing research articles to identify key proteins that physically or functionally interact with REST, as well as those known to influence its stability, localization, or transcriptional activity. This was followed by a STRING database (Szklarczyk et al., 2022) analysis for REST and its interactors, with 50 interactors being listed with a high confidence (>0.750). This yielded a list which was analysed on Cytoscape, using Cytoscape’s cytoHubba (Chin et al., 2014) plugin, which ranks nodes based on network features using curated STRING interaction data. The interactors were prioritised based on topological measures (Degree, Closeness and Betweenness), and the top-ranked proteins were then corroborated with evidence from literature for their relevance to REST functional pathways.

### 2.6 Docking of REST with its interactors

To critically analyse the functional impact of the five SNPs on the human REST protein, both protein-protein and protein-DNA docking analysis were performed. Docking was conducted between the wild-type REST and its five mutant forms with each of the selected five interactors (HDAC1, HDAC2, KDM1A, RBBP7 and SIN3A) and the Re1 motif, enabling evaluation of to what extent the mutations are affecting REST’s function as a transcriptional repressor.

**FIGURE 2:**
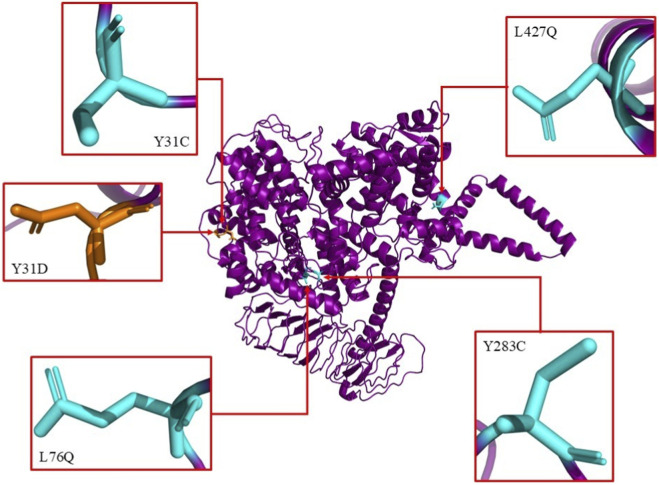
Illustration of the predicted structure of human REST protein with the 5 most deleterious SNP mutations predicted across various SNP analysis tools. The 5 most deleterious SNP mutations were predicted to be Y31C, Y31D, L76Q, Y283C and L427Q.

**FIGURE 3:**
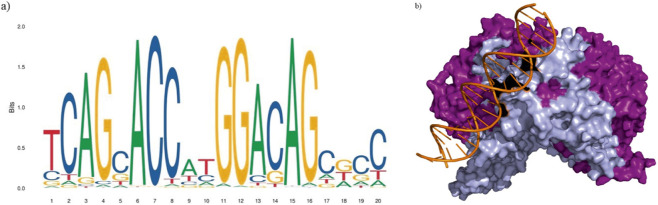
Consensus sequence of Re1 motif. **(a)** Sequence logo of the 21-bp consensus sequence of Re1 motif (5’-TCAGCACCATGGACAGAGCC-3’). **(b)** Predicted structure of human REST protein (purple) docked with the Re1 motif (orange) found upstream of its target genes. The zinc finger domains (residues 2-489) of the REST protein are represented in lilac.

In silico mutations were induced on the refined model of wild-type REST to obtain the mutated REST protein structures (Figure 2) using SwissPDB Viewer, a tool for visualizing and manipulating 3D structures of proteins and nucleic acids (Waterhouse et al., 2018). AlphaFold Protein Structure Database (Jumper et al., 2021; Varadi et al., 2023) was used to obtain the 3D structures of the five protein interactors. For the Re1 motif, its sequence was obtained from the JASPAR database which provided two entries whose matrix IDs were MA0138.1 and MA0138.2 respectively (Sandelin et al., 2003). Compared to MA0138.1, which is a 19 bp sequence, MA0138.2 is a 21 bp sequence and has higher frequency values at each position in the position frequency matrix. So, the 21 bp sequence (5′-TTC AGC ACC ATG GAC AGC GCC-3′) was chosen as it better represents the binding preferences of REST with higher statistical support (Figure 3a). To model the 3D structure of the Re1 motif, PyMOL software was utilized using the FASTA format of the DNA sequence (PYMOL, 2025) (Figure 3b).

HADDOCK 2.4 web server (Honorato et al., 2024) was employed to perform the docking between the wild and mutant REST and the five protein interactors, as well as the Re1 motif. Active residues of the REST variants and the protein interactors were identified using the ScanNet web server (Tubiana et al., 2022), which predicts residue-level binding probabilities from 3D structural features. For each protein, the residue with the highest predicted probability was selected and provided as the active residue input for docking HADDOCK outputs clusters of docking models grouped by structural similarity and ranked by the HADDOCK score, with lower scores indicating more favorable interactions. Each cluster is also assigned a z-score reflecting the deviation of its score from the overall mean; more negative z-scores denote more significant docking solutions.

### 2.7 Molecular dynamics simulation

#### 2.7.1 REST and mutant variants (unbound simulation)

To investigate the structural dynamics and global stability of REST and its five selected mutant variants (Y31C, Y31D, L76Q, Y283C, and L427Q), MD simulations were conducted using GROMACS version 2023.1. The simulation followed the “Lysozyme in Water” protocol as described by Abraham et al. (Abraham et al., 2015), adapted to suit the REST protein system. The aim was to identify atomic-level changes in flexibility, compactness, and thermodynamic properties that result from single-point missense mutations in REST.

Preprocessing involved removing nonstandard residues and heteroatoms from the wild-type REST PDB file. The system was prepared using the OPLS/AA force field, where the protein was embedded in a cubic simulation box with a 1.0 nm buffer, solvated using the SPC216 water model and electrostatically neutralized with Cl^−^ counter-ions.

Energy minimization was then performed using the steepest descent algorithm, followed by NVT equilibration using a modified Berendsen thermostat, and NPT equilibration using the Parrinello-Rahman barostat. Each system then underwent a 200 ns production MD simulation under periodic boundary conditions.

Trajectory analyses were performed using standard GROMACS utilities. Root mean square deviation (RMSD), root mean square fluctuation (RMSF), radius of gyration (Rg), and potential energy (PE) profiles were generated to compare the structural behavior and stability of wild-type REST with its mutant counterparts. These analyses allowed for the quantification of rigidity, conformational drift, folding compactness, and energy favorability across the variants.

#### 2.7.2 REST-Re1 motif complex simulation (bound simulation)

To specifically assess how the Y283C mutation affects REST’s DNA-binding dynamics, an additional MD simulation was carried out for the REST-Re1 motif complex. Both the wild-type REST-Re1 complex and the mutant 4 (Y283C)-Re1 complex were modeled and simulated for this purpose. This was done by processing and standardizing the preparation of the system using CHARMM-GUI (Jo et al., 2008).

The initial system preparation was performed using the CHARMM-GUI Solution Builder (Lee et al., 2015; Brooks et al., 2009). The CHARMM36 force field was employed for topology generation. The REST-DNA complex was processed in the PDB Reader module to assign protonation, rebuild missing atoms, and convert the system into a GROMACS-compatible format. The complex was placed in a cubic TIP3P water box with a 10 Å buffer, and 0.15 M KCl was added using the Monte Carlo ion-placement procedure to achieve physiological ionic strength and charge neutrality.

Subsequently, CHARMM-GUI generated input files for steepest descent energy minimization, NVT and NPT equilibration, and unrestrained production dynamics. A 100 ns production MD simulation was then performed for both the wild-type REST-Re1 and mutant 4-Re1 systems.

Trajectory data were analyzed using GROMACS utilities to compute RMSD, RMSF, Rg, and PE profiles. These comparative analyses helped characterize how the Y283C mutation impacts the stability, compactness, and conformational integrity of REST in its DNA-bound state. Special attention was given to changes in the zinc finger domain and Re1 interface, as these are essential for REST’s transcriptional repressor function.

## 3 Results and discussion

### 3.1 REST SNP landscape: identification of pathogenic variants

The transcriptional repressor REST protein was found to be a protein with a sequence length of 1097 aa. dbSNP results showed 12974 SNPs that have been discovered in REST, with 938 of them being missense mutations. All 938 were compiled in a list and used for SNP analysis.

### 3.2 Highly accurate REST protein structure

AlphaFold3 predicted five structural models and on comparison of their per-residue pLDDT confidence scores (Supplementary Figures S1a–e), it showed that all models exhibited highly similar overall folds and nearly identical pLDDT profiles, indicating minimal structural variability across them. High-confidence scores (pLDDT >80) were observed across the structured regions of REST, including the N-terminal repression region and the zinc-finger domains. While, several loop regions displayed comparatively lower pLDDT values, but this pattern was reproducible across all five models. Further structural validation and quality assessment were performed using ERRAT and Ramachandran plot analysis. Among the five models, Model 1 was identified as the most reliable, displaying a superior ERRAT score of 64.1873 and 92.9% of residues in the favored region of the Ramachandran plot, with 0% in the disallowed region (Table 1). This indicated that Model 1 had better stereochemical quality and fewer structural distortions than the other models. The selected Model one contained well-defined secondary structure elements, particularly within the eight zinc-finger DNA-binding motifs and the core repression domain, both of which are crucial for REST’s role in transcriptional repression.

**TABLE 1:** Validation of the structural quality of the predicted 3D structure of REST protein from AlphaFold3 using ERRAT score and Ramachandran Plot Data from PROCHECK tool (Accessed from SAVES v6.1 made by UCLA-DOE LAB).

			Ramachandran plot				
Protein	Model	ERRAT score	Favoured (%)	Additional (%)	Generous (%)	Disallowed (%)	Total accepted (%)
REST	Model 1	64.19	92.9	7.1	0	0	100
	Model 2	57.39	90	10	0	0	100
	Model 3	56.05	91.6	8.3	0.1	0	100
	Model 4	47.33	91.7	8.3	0	0	100
	Model 5	55.36	91.8	8.2	0	0	100
	Model 1 (refined)	80.94	94.2	5.8	0	0	100

**FIGURE 4:**
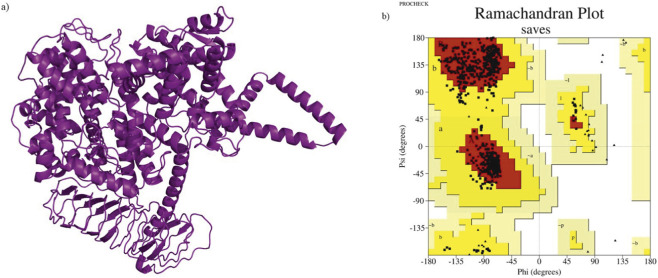
Structural Assessment of REST Model **(a)** 3D structure of the REST protein generated using AlphaFold3, visualized using PyMOL. **(b)** Ramachandran plot generated by PROCHECK for the homology model, depicting the distribution of backbone dihedral angles (phi and psi). The plot shows 92.9% of residues in the most favored regions (red) and 7.1% in additionally allowed regions (yellow), with no residues in generously allowed or disallowed regions, indicating the model's high stereochemical quality and structural reliability.

Model one was subjected to refinement using GalaxyRefine and the refined model, which was visualised using PyMOL (Figure 4a), exhibited significant improvements in structural quality, as observed in the validation metrics. The ERRAT score increased from 64.1873 to 80.9392, indicating reduced overall error and improved backbone conformation. Furthermore, Ramachandran plot analysis showed an increase in the favored region percentage from 92.9% to 94.2%, with no residues in the disallowed region (Figure 4b). The additional allowed region decreased from 7.1% to 5.8%, demonstrating an overall enhancement in structural reliability.

These improvements confirmed that Model 1 (Refined) represented the most accurate and stable structural model of REST, making it well-suited for subsequent analyses such as mutation modeling, molecular docking with interactors (HDAC1, HDAC2, KDM1A, SIN3A, and RBBP7), and protein-DNA interaction studies with the Re1 motif. The refined structure provided a robust template for investigating the effects of pathogenic mutations and their potential role in disrupting REST’s transcriptional repression function, leading to dysregulation of downstream gene targets. However, the model still carries inherent limitations, as no full-length experimental structure of REST is available for direct comparison and the experimentally resolved fragments that do exist correspond only to the zinc-finger domains or small isolated domains that do not overlap with most of the modeled regions. Consequently, direct benchmarking of the domain-domain arrangement of the predicted model against experimental data was not feasible. In addition, regions with lower pLDDT confidence, particularly in flexible loops and linkers, retain greater structural uncertainty. Accordingly, the docking outcomes should be viewed in light of these confidence variations within the predicted structure.

### 3.3 Pathogenic REST variants

To identify functionally impactful missense SNPs in the REST protein, a systematic computational filtering approach was employed. Out of the 938 available missense SNPs, only those predicted to be deleterious and structurally destabilizing were shortlisted for further analysis. The initial screening was performed using SIFT, which predicts whether an amino acid substitution affects protein function based on sequence homology and conservation. Only SNPs with a SIFT score of 0, indicative of a highly deleterious effect, were retained, reducing the total number from 938 to 429. Further classification using PolyPhen-2 (HumVar model) categorized these 429 SNPs into three groups: 152 were classified as probably damaging (score >0.950), 82 were possibly damaging (score between 0.500 and 0.950), and 195 were benign (score <0.500). For further structural and stability assessments, only the 152 probably damaging SNPs with a PolyPhen-2 score >0.950 and a SIFT score of 0 were considered, and to ensure the highest confidence in deleterious predictions, the top 89 SNPs with a PolyPhen-2 score greater than 0.997 were shortlisted. The top 89 SNPs and their respective scores across the five tools are provided in the Supplementary Table 1.

The five most deleterious SNPs, Y31C, Y31D, L76Q, Y283C, and L427Q, were analyzed further using PhD-SNPg, mCSM, and DynaMut2 to assess their impact on REST stability. All five SNPs were classified as pathogenic by PhD-SNPg, with scores ranging from 0.903 to 0.956 (Table 2). Structural stability analysis using mCSM and DynaMut2 revealed significant destabilization, as indicated by the negative ΔΔG (kcal/mol) values, confirming that these mutations contribute to structural disruption. Among these, Y31D exhibited the highest destabilization, with a DynaMut2 ΔΔG of −3.36 kcal/mol and an mCSM ΔΔG of −3.08 kcal/mol, suggesting severe perturbation within REST’s functional domain. These results strongly suggest that these missense mutations significantly destabilize REST, potentially impairing its function in transcriptional repression.

**TABLE 2:** Analysis of the five high-confidence deleterious SNPs in the REST protein identified through *in silico* tools.

SNP	Amino acid position and substitution	SIFT	POLYPHEN2 (HumVar)	PhD-SNPg	mCSM	Dynamut2 DDG value Kcal/mol
rs967744114	Y31C	0; deleterious	1; probably damaging	0.953; pathogenic	−2.04; highly destabilizing	−2.65; destabilizing
rs778338685	Y31D	0; deleterious	0.999; probably damaging	0.953; pathogenic	−3.08; highly destabilizing	−3.36; destabilizing
rs1221861537	L76Q	0; deleterious	1; probably damaging	0.956; pathogenic	−2.381; highly destabilizing	−2.6; destabilizing
rs1014438653	Y283C	0; deleterious	1; probably damaging	0.956; pathogenic	−1.524; destabilizing	−2.12; destabilizing
rs1179513496	L427Q	0; deleterious	1; probably damaging	0.903; pathogenic	−1.848; destabilizing	−1.81; destabilizing

### 3.4 Conserved residues in REST protein

The of REST protein sequences from ten primate species revealed strong evolutionary conservation, particularly in domains essential for transcriptional repression. The percent identity matrix showed >94% similarity across species, with nearly 99% identity between humans and close relatives like chimpanzees and bonobos.

Four key residues, Y31, L76, Y283, and L427, were strictly conserved, underscoring their likely functional importance. Y31C, Y31D, and L76Q are located in the N-terminal repressor domain (residues 1–73), which recruits cofactors such as CoREST, SIN3A, and HDACs (Imm et al., 2000). Their conservation suggests a crucial role in maintaining REST-cofactor interactions, and mutations here may disrupt repression of genes linked to ECM accumulation and cell proliferation.

Y283C and L427Q are situated within the zinc finger DNA-binding family domains (residues 2–489), critical for DNA recognition. Mutations at these conserved sites may weaken REST’s ability to repress genes involved in fibrotic and proliferative signaling, potentially contributing to fibroid development.

Overall, the high conservation of these residues supports their structural and functional indispensability. Mutations at these sites may impair REST’s repressive capacity, suggesting a possible association with fibroid-related transcriptional changes and identifying them as hypothesis-generating candidates for future therapeutic investigation.

### 3.5 REST interactome topology: SIN3A and HDAC1/2 emerge as key hubs

**FIGURE 5:**
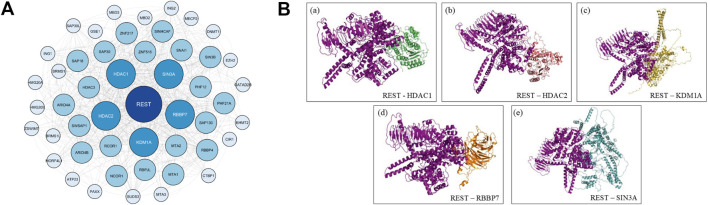
Network analysis and docked image. **(A)** Network Based Analysis of REST Protein Interactions Using STRING Database. Protein-protein interaction networks of REST were generated from the STRING database and visualized using Cytoscape. **(B)** Illustrations of the predicted structure of human REST protein docked with the 5 highest scoring protein interactors from STRING database. The 5 highest scoring protein interactors of the REST protein were HDAC1, HDAC2, KDM1A, RBBP7 and SIN3A.

The STRING database yielded 50 interactors which was then viewed as a functional network image using Cytoscape (Figure 5A). Using Cytoscape’s cytoHubba plugin, the network was ranked based on topological parameters, including Degree, Betweenness, and Closeness centrality measures, to identify key regulatory hubs.

This analysis identified several high-ranking REST interactors, including HDAC1, HDAC2, SIN3A (Huang et al., 1999; Grimes et al., 2000), KDM1A (LSD1) (Shi et al., 2004), and RBBP7 (Ooi and Wood, 2007). HDAC1 and HDAC2 showed the highest centrality scores, with SIN3A also ranking prominently, consistent with their established roles in REST-associated co-repressor complexes (Ooi and Wood, 2007; Bithell, 2011; Jayaprakash et al., 2020). KDM1A and RBBP7 likewise appeared as key nodes, supporting their involvement in REST-mediated chromatin regulation.

The network analysis supports previous experimental findings that REST dysfunction may contribute to altered gene expression in uterine fibroids. The strong association of REST with these regulatory proteins suggests that its interaction network plays a key role in maintaining transcriptional balance. This network-based approach provides a systems-level view of REST’s interactome and highlights several potential molecular targets for further investigation. Future studies should aim to validate these interactions through protein-protein interaction assays and investigate their role in REST-mediated transcriptional regulation in uterine fibroids.

### 3.6 Destabilizing mutations weaken protein-protein and DNA binding

Wild and mutant REST was docked with the selected five protein interactors (HDAC1, HDAC2, KDM1A, RBBP7 and SIN3A) and its DNA interactor, the Re1 motif using the HADDOCK 2.4 web server (Figure 5B). The wild-type Z-scores served as benchmarks for comparing mutant interaction strengths, where less negative Z-scores indicated weaker binding. Table 3 summarizes the Z-scores and HADDOCK scores for the docking analysis performed.

**TABLE 3:** Z-scores and HADDOCK scores for the interaction of REST and mutants with their various protein interactors and Re1 motif from HADDOCK docking simulations. The difference between the Z-scores of each of the mutant-complexes and their respective wild-type complexes is given in brackets.

REST	HDAC1		HDAC2		KDM1A		RBBP7		SIN3A		Re1 motif		Avg. Z-score
	Z-score	Haddock score	Z-score	Haddock score	Z-score	Haddock score	Z-score	Haddock score	Z-score	Haddock score	Z-score	Haddock score	
Wild	−1.5	−69.7 ± 1.3	−2.1	−62.8 ± 9.2	−2.2	−87.5 ± 7.4	−1.5	−82.6 ± 0.5	−2.4	−64.4 ± 7.8	−2.0	−22.8 ± 3.1	−1.95
Mutant 1	−1.9 (−0.4)	−77.9 ± 2.4	−1.8 (+0.3)	−55.2 ± 11.3	−1.6 (+0.6)	−76.5 ± 2.8	−1.9 (−0.4)	−85.3 ± 1.4	−1.6 (+0.8)	−64.4 ± 7.8	−1.3 (+0.7)	−20.3 ± 13.6	−1.68 (+0.27)
Mutant 2	−2.4 (−0.9)	−80.1 ± 19.1	−1.9 (+0.2)	−74.4 ± 10.6	−1.9 (+0.3)	−84.9 ± 3.9	−2.2 (−0.7)	−85.7 ± 1.6	−2.1 (+0.3)	−64.4 ± 7.8	−1.3 (+0.7)	−25.3 ± 4.1	−1.97 (−0.02)
Mutant 3	−2.1 (−0.6)	−73.6 ± 12.6	−1.7 (+0.4)	−61.8 ± 17.6	−1.5 (+0.7)	−66.4 ± 7.9	−1.9 (−0.4)	−86.9 ± 4.0	−1.6 (+0.8)	−64.4 ± 7.8	−1.7 (+0.3)	−22.2 ± 7.5	−1.75 (+0.20)
Mutant 4	−1.8 (−0.3)	−69.1 ± 11.3	−1.6 (+0.5)	−69.9 ± 5.6	−1.5 (+0.7)	−75.3 ± 11.4	−2.0 (−0.5)	−90.3 ± 2.6	−1.4 (+1.0)	−53.5 ± 3.3	−1.3 (+0.7)	−21.9 ± 7.0	**−1.60 (+0.35)**
Mutant 5	−2.0 (−0.5)	−70.6 ± 10.9	−1.7 (+0.4)	−62.1 ± 6.8	−1.4 (+0.8)	−73.0 ± 10.0	−2.0 (−0.5)	−86.3 ± 2.0	−1.4 (+1.0)	−55.9 ± 6.8	−1.7 (+0.3)	−22.7 ± 10.5	−1.70 (+0.25)

Bold values indicate the least favourable (least negative average Z-score) interactions among the wild-type and mutant complexes.

Upon analysis of the Z-scores for protein-protein docking analysis, it was found that all the five mutant complexes with SIN3A had a lesser negative Z-score compared to the wild-SIN3A complex, which had a score of −2.4. This suggests that the selected mutations do diminish the interaction profile between REST and SIN3A. Amongst the five mutations, mutant 4 (Y283C) and mutant 5 (L427Q) complexes with SIN3A particularly had the greatest positive differential in Z-score compared to that of the wild-type. Considering the role of SIN3A in the REST corepressor complex it can be inferred that SNP mutations such as Y283C and L427Q could hinder the recruitment of SIN3A by REST, thereby preventing the formation of HDAC1/HDAC2 complex and histone deacetylation, leading to increased gene expression associated with uterine fibroids.

Similarly, Y283C showed the highest positive Z-score differentials with HDAC2 (+0.5) and KDM1A (+0.7), indicating reduced affinity relative to their wild-type complexes. These results suggest that Y283C is the most broadly destabilizing mutation across multiple interactors.

On the contrary, the docking analysis revealed that all the five mutant complexes with HDAC1 and RBBP7 had greater binding affinity between them, compared to the corresponding wild-type complex as they had a more negative Z-score than their wild-type complexes. This finding illustrates that while certain deleterious mutations cause a decrease in binding affinity with key interactors, the same mutations can cause an increase in binding affinity with other interactors simultaneously. Further studies must be conducted to examine the resultant effect of these changes in the interaction profile between REST and its interactors in the corepressor complex.

For the protein-DNA docking analysis, the wild and mutant REST protein was docked with the 21 bp Re1 motif. It was found that the wild-type REST protein had the strongest binding interaction with the motif, indicated by a Z-score of −2.0. Mutants 1, 2 and 4 exhibited the least binding affinity with Re1, each complex having a Z-score of −1.3.

While the docking trends provide a consistent comparative view of how each mutation affects REST’s interactions, the HADDOCK output available for analysis is limited to global energetics and top model rankings. As a result, the present docking results indicate relative changes in binding affinity but do not permit definitive mechanistic conclusions at the interface level due to this inherent resolution constraint.

Overall, mutant 4 consistently demonstrated a poor binding affinity with its protein and DNA interactors compared to the other mutants. Its complexes exhibited an average Z-score of −1.60, the least negative among all mutant complexes, indicating the weakest interactions. Given this observation, mutant 4 was selected for further structural investigation. MD simulations were conducted on the wild-type REST and mutant 4 in complex with the Re1 motif, the primary DNA element recognized by REST, to uncover how the mutation alters REST’s conformation, stability, and DNA-binding dynamics. This targeted approach allowed for a focused comparison of functional consequences at the level of REST-Re1 interaction. Overall, Y283C showed the weakest interactions across protein and DNA partners, with an average Z-score of −1.60, the least negative among mutants. Based on this, Y283C was selected for further investigation using MD simulations of wild-type and mutant REST in complex with the Re1 motif to assess effects on conformation, stability, and DNA-binding dynamics.

### 3.7 Conformational dynamics reveal mutation-specific destabilization

#### 3.7.1 Unbound mutants display distinct stability profiles

To evaluate the impact of the selected deleterious SNPs on the structural and dynamic behaviour of the REST protein, 200 ns MD simulations were performed on the wild-type and its five mutant variants. The trajectories were analysed based on root mean square deviation (RMSD), root mean square fluctuation (RMSF), radius of gyration (Rg), and potential energy (PE). Table 4 summarizes the average values for each of the parameters for the wild type REST and its five mutants.

**TABLE 4:** Comparative molecular dynamics-derived parameters for REST wild type and five mutant proteins.

REST protein	Average RMSD (nm)	Average RMSF (nm)	Average radius of gyration (nm)	Average potential energy (MJ/mol)
Wild type	0.85	0.33	3.48	−3708.84
Mutant 1	0.79	0.34	3.44	−3703.79
Mutant 2	0.83	0.23	3.42	−3709.54
Mutant 3	0.89	0.30	3.42	−3709.51
Mutant 4	0.85	0.27	3.51	−3709.45
Mutant 5	1.01	0.35	3.49	−3709.69

The table presents average Root Mean Square Deviation (RMSD), Root Mean Square Fluctuation (RMSF), Radius of Gyration (Rg), and Potential Energy values for the REST wild-type protein and its five mutants across the simulation trajectory. These metrics assess the structural stability, flexibility, compactness, and energetic favorability of each variant.

**FIGURE 6:**
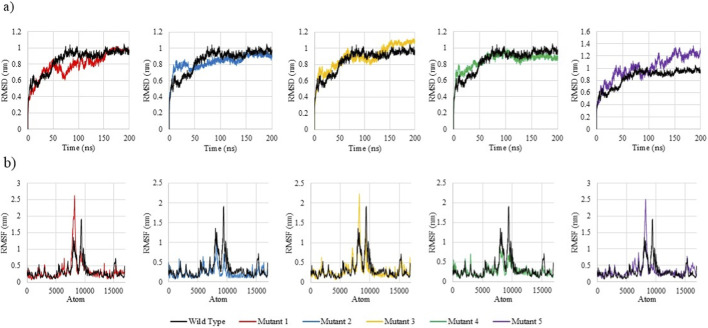
Comparative RMSD and RMSF profiles of Wild-Type REST and its 5 mutants over 200 ns MD simulations. **(a)** RMSD profiles showing time-dependent structural deviations of wild-type REST and its five mutants over 200 ns MD simulations. **(b)** RMSF profiles depicting residue-wise flexibility patterns of the wild-type and mutant proteins.

The RMSD profiles (Figure 6a) revealed that the wild-type REST protein achieved equilibrium early and maintained a stable conformation throughout, with an average RMSD of 0.85 nm, indicative of a stable and well-folded structure. Mutant 1 (Y31C) had the lowest average RMSD (0.79 nm), suggesting a slightly more rigid conformation. This rigidity could hinder conformational dynamics necessary for binding with the Re1 motif or co-repressors. Mutants 2 (Y31D) and 3 (L76Q) showed only subtle deviations from the wild type, suggesting limited structural impact. Mutant 4 (Y283C) showed wild-type–like RMSD (0.85 nm), while mutant 5 (L427Q) exhibited the highest average RMSD (1.01 nm) with prominent temporal fluctuations, indicating greater global drift.

The RMSF profiles (Figure 6b) illustrated that the wild type had an average RMSF of 0.33 nm, with moderate fluctuations primarily localized to its loop regions. Mutants 2 (Y31D) and Mutant 4 (Y283C) exhibited the lowest RMSFs (0.23 nm and 0.27 nm). This striking reduction in residue fluctuation suggests a more rigid conformation. This rigidity could compromise REST’s transcriptional repression function. Conversely, mutant 5 (L427Q) showed increased motion (0.35 nm), destabilizing key functional domains. Notably, significant RMSF spikes were observed near atomic residue positions ∼8200 in Mutants 1, 3, and 5, indicating a region sensitive to structural disturbances, even from distant mutations. This suggests possible long-range destabilization affecting folding or chromatin interaction.

**FIGURE 7:**
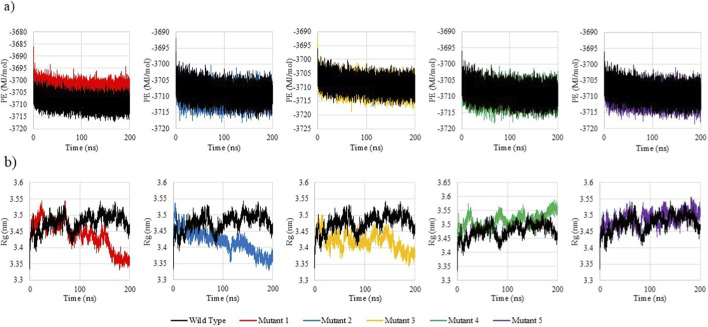
Comparative PE and Rg profiles of Wild-Type and 5 REST mutants over 200 ns MD simulations. **(a)** Potential energy (PE) profiles illustrating the energetic stability of wild-type REST and its five mutants during 200 ns MD simulations. **(b)** Radius of gyration (Rg) profiles showing time-dependent changes in structural compactness of the wild-type and mutant proteins.

The Rg analysis (Figure 7a) demonstrated that the wild type REST maintained a compact structure (avg. Rg = 3.476 nm). Mutants 2, 3 and 1 had slightly lower Rg values at 3.419 nm, 3.421 nm and 3.439 nm, respectively, suggesting marginally more compact folds. Interestingly, mutants 2 and 3 also demonstrated late-stage compaction in the final ∼50 ns, possible due to internal strain or loss of conformational flexibility. Mutant 1 showed a steady compaction trend across the simulation. In contrast, mutants 4 and 5 exhibited elevated Rg values, with mutant 4 having the highest at 3.512 nm, suggesting persistent structural relaxation. Since Y283 lies in the zinc finger domain, such unfolding may disrupt REST’s ability of recognition of the Re1 motif and chromatin repression.

The PE plots (Figure 7b) show that the wild-type REST protein had a stable, average PE of −3708.84 MJ/mol. Mutant 1 had a relatively unfavourable energy (−3703.79 MJ/mol), suggesting subtle internal strain within the. Conversely, all other mutants exhibited marginally more negative PE values than the wild-type, with Mutant 5 showing the most favourable energy (−3709.70 MJ/mol).

Taken together, the unbound MD simulations revealed distinct structural effects across the five REST mutants. Mutant 5 (L427Q) showed increased global flexibility, whereas mutant 2 (Y31D) was markedly more rigid. Mutant 4 (Y283C) emerged as the most perturbed variant: despite wild-type–like RMSD, it displayed the highest Rg and one of the lowest RMSF values, consistent with a less compact yet locally rigid fold. Docking further indicated weakened binding of Y283C to key co-repressors (HDAC2, SIN3A, KDM1A) and the Re1 motif, in line with its location in the zinc finger DNA-binding domain and the predicted disruption of both protein–protein and protein–DNA contacts. On this basis, Y283C was selected for additional MD simulations in complex with the Re1 motif to directly assess its impact on DNA-binding stability and REST’s regulatory function in uterine fibroids.

#### 3.7.2 Mutant REST with Re1 DNA binding stability

MD simulations revealed significant structural and energetic differences between the wild-type REST-Re1 complex and the Y283C mutant (Mutant 4), underscoring the mutation’s disruptive effect on REST’s repressive function. As summarized in Table 5, the mutant complex showed a notable increase in potential energy and a subtle decrease in compactness, suggesting impaired structural stability.

**TABLE 5:** Comparison of REST-Re1 complex dynamics between wild-type and Mutant 4 REST.

REST-Re1 complex	Average RMSD (nm)	Average RMSF (nm)	Average radius of gyration (nm)	Average potential energy (kJ/mol)
Wild type	0.95	0.39	3.56	−4442638.64
Mutant 4	1.07	0.40	3.51	−3165625.22

Average values of RMSD, RMSF, radius of gyration, and Potential Energy for the REST-Re1, complex involving wild-type REST, and Mutant 4. The data highlight the structural and energetic impact of the mutation on REST’s interaction with the Re1 DNA, motif.

**FIGURE 8:**
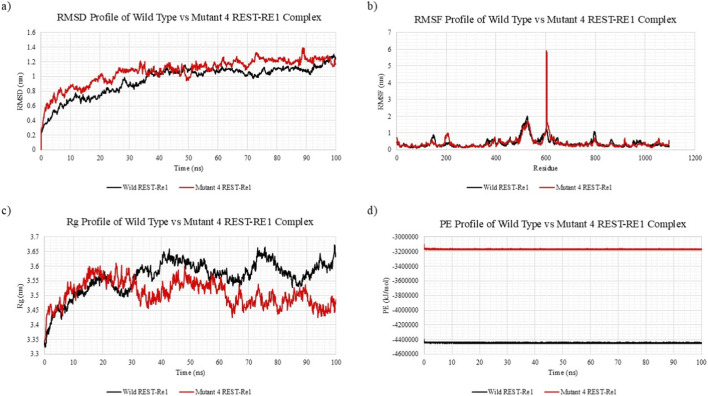
Molecular Dynamics Simulation Profiles Comparing Wild-Type (Black) and Mutant 4 - Y283C (Red) REST-Re1 Complexes over 200 ns. **(a)** Root mean square deviation (RMSD) analysis shows that the mutant complex exhibits higher structural deviations compared to the wild-type, indicating reduced conformational stability. **(b)** Root mean square fluctuation (RMSF) profile reveals a pronounced spike near residue 600 in the mutant complex, suggesting long-range destabilizing induced by the Y283C mutation, despite comparable average fluctuations to the wild type. **(c)** Radius of gyration (Rg) plots indicate that while the mutant complex appears marginally more compact on average, it undergoes more pronounced temporal fluctuations, implying localized instability. **(d)** Potential energy (PE) profiles demonstrate a significant thermodynamic destabilization in the mutant complex, with a much higher average energy compared to the wild-type, suggesting reduced binding and functional impairment.

RMSD analysis (Figure 8a) demonstrated that the wild-type REST-Re1 complex reached equilibrium early and maintained a stable trajectory, with an average RMSD of 0.95 nm over the 200 ns simulation. In contrast, the Y283C mutant exhibited elevated and sustained deviations, with an average RMSD of 1.07 nm, indicating increased flexibility and reduced structural integrity that may compromise stable engagement of the Re1 motif.

RMSF analysis (Figure 8b) indicated that mean fluctuations were similar between wild-type (0.39 nm) and mutant (0.40 nm), but several regions in the mutant exhibited markedly higher mobility. Residues within and near the zinc finger DNA-binding domains, including the region surrounding Y283, showed increased flexibility, suggesting that the tyrosine-to-cysteine substitution perturbs local hydrogen bonding and metal coordination and destabilizes the DNA-binding core.

A pronounced RMSF spike was observed near residue 600 in the mutant complex, with fluctuations approaching 6 nm. Although residue 600 is distant from the mutation site, this behaviour suggests a long-range destabilization affecting distal loops or linkers important for coordinating inter-domain alignment and co-repressor interactions, potentially impairing assembly of a functional repressive complex.

The radius of gyration (Rg) analysis (Figure 8c) showed that while the mutant complex maintained a slightly lower average Rg (3.51 nm vs. 3.56 nm wild-type), it exhibited more pronounced temporal fluctuations in Rg values. This pattern of transient expansions and contractions is characteristic of increased internal flexibility or partial unfolding, particularly in regions essential for DNA and cofactor interaction.

Energetic analysis (Figure 8d) further supported the destabilizing impact of Y283C. The wild-type REST–Re1 complex displayed a low, stable potential energy of −4,442,638 kJ/mol, whereas the mutant complex showed a markedly higher (less favourable) potential energy of −3,165,625 kJ/mol, consistent with a thermodynamically less stable conformation and reduced DNA-binding affinity inferred from docking.

Global differences in RMSD, RMSF, Rg and potential energy among unbound REST simulations were modest and largely within expected thermal fluctuations, so these metrics were interpreted qualitatively and alongside docking data rather than as stand-alone indicators of destabilization (Table 4). By contrast, the REST–Re1 bound complex showed more distinct perturbations for Y283C, including the pronounced RMSF spike near residue ∼600 and substantially higher potential energy relative to the wild-type complex (Figure 8; Table 5), supporting a model of localized functional disruption at the DNA-binding interface rather than global unfolding.

The MD simulations were designed as qualitative structural comparisons rather than free-energy or ensemble-averaged calculations. Accordingly, we employed single, long equilibrated trajectories for each system (200 ns for REST and mutant variants and 100 ns for the REST–Re1 simulations), as is standard in comparative bound-state MD analyses. Convergence was reached rapidly, and the RMSD, Rg and potential energy profiles for all systems stabilized early and fluctuated only within narrow ranges thereafter (Figures 6–8). Under these conditions, additional replicas are unlikely to expand sampling to a different conformational basin or materially alter the qualitative interpretation, as the trends were reproducible across independent observables and across both the unbound and bound simulations. This approach is consistent with widely adopted MD practice for systems in which the objective is to compare structural trends at equilibrium rather than to quantify rates, rare events or free energies.

Although the present work identifies structural and dynamic effects associated with REST mutations, these findings have not yet been validated experimentally. As this study is computational in nature and focused on predicting structural consequences and prioritizing candidate variants, biochemical or cellular assays fall outside the scope of the current investigation. Nevertheless, future studies that evaluate the impact of key variants, particularly Y283C, using experimental approaches will be essential to confirm and further characterize the regulatory and functional implications predicted here. Additionally, while the co-occurrence of these mutations in patients and their potential combined effect on REST function remain unclear, investigating combinatorial or additive mutational impacts on REST stability and repression activity represents an important future direction and could provide valuable insight into the biological relevance of such interactions.

The MD simulations highlight substantial structural and energetic consequences of the Y283C mutation in the REST–Re1 complex. Elevated RMSD, localized RMSF spikes (notably near residues 283 and 600), enhanced Rg fluctuations, and a marked increase in potential energy together indicate reduced conformational precision and functional stability in the mutant complex. These perturbations likely weaken REST’s ability to maintain stable Re1 binding and assemble its repressive machinery, with the extreme mobility around residue 600 emerging as a potential dynamic signature of REST inactivation. Overall, the data support Y283C as a mechanistically important driver of REST dysfunction that may contribute to aberrant activation of fibroid-associated targets such as GPR10.

## 4 Conclusion

Uterine fibroids (leiomyomas) are among the most prevalent benign tumors affecting women of reproductive age, yet their molecular etiology remains incompletely understood, rendering them a persistent clinical and biological enigma. While traditionally considered hormonally driven, emerging evidence points to a significant role of additional molecular contributors to their pathogenesis. One of the most compelling recent discoveries in this context is the implication of the transcriptional repressor REST as a central regulatory node in fibroid biology. REST functions as a master chromatin remodeler, silencing neuronal and growth-promoting genes in non-neuronal tissues by binding to the Re1 DNA motif and recruiting corepressor complexes. Disruption of this pathway has the potential to lift essential transcriptional constraints, triggering aberrant gene expression programs that contribute to fibroid development and progression.

In this study, we employed an integrative *in silico* strategy to dissect the structural and functional consequences of specific missense mutations in REST. Through evolutionary conservation analysis, structural modeling, docking simulations, and molecular dynamics, we characterized five deleterious variants, Y31C, Y31D, L76Q, Y283C, and L427Q, found to compromise key features of REST’s regulatory architecture. Among them, the Y283C mutation emerged as particularly disruptive. Located within a zinc finger motif essential for DNA recognition, Y283C induced profound structural destabilization, weakened REST’s binding affinity for the Re1 motif, and impaired its interactions with corepressors such as HDAC2, SIN3A, and KDM1A. These structural deficits were compounded by molecular dynamics simulations, which revealed enhanced flexibility, local unfolding, and a thermodynamic penalty that further undermined complex stability. Importantly, the effects of the mutation extended beyond the local environment, propagating conformational shifts across the protein that likely interfere with domain alignment and transcriptional control.

These mechanistic insights take on added significance in light of REST’s role in repressing uterine growth factors such as GPR10, whose overexpression promotes PI3K/AKT/mTOR pathway activation, a known driver of fibroid proliferation. The observed reduction in REST silencing function suggests a plausible mechanistic hypothesis in which single-point alterations may contribute to changes in chromatin organization and could influence pro-fibrotic transcriptional programs. Notably, the residues altered by these mutations are highly conserved across ten primate species, underscoring their evolutionary indispensability and highlighting the selective vulnerability of the REST protein to even subtle amino acid substitutions. The fact that such minimal sequence changes elicit wide-ranging effects on structural stability, DNA binding, and cofactor interaction suggests that certain regions of REST are finely tuned for regulatory precision, making them particularly susceptible to functional derailment when mutated.

Together, these findings provide a rare structural perspective on how minor genetic perturbations can destabilize a major transcriptional repressor, with consequences that reverberate through critical regulatory systems. REST therefore emerges as a factor of interest in fibroid biology, whose perturbation may be relevant to early molecular alterations, warranting further experimental investigation. While the computational findings provide structural insight into how variants such as Y283C may influence REST behavior, these results are predictive and do not establish direct causality in uterine fibroid pathogenesis. Instead, they generate hypotheses for future experimental validation and motivate additional wet-lab studies to confirm the functional relevance of these variants.

The REST interactome, its zinc finger domains, and the Re1 interface now stand out as critical focal points for deeper investigation, each representing a potential point of fragility within the molecular framework that maintains uterine homeostasis. By tracing the path from conserved sequence alterations to macromolecular dysfunction, this study reinforces the significance of high-resolution molecular interrogation in uncovering hidden drivers of complex, non-malignant pathologies.

## Data Availability

The raw data supporting the conclusions of this article will be made available by the authors, without undue reservation.
