# Supplementary figures and images for: REST missense mutations reveal disrupted Re1 motif binding and co-repressor interactions in uterine fibroids
Source: Front Bioinform. 2026 Jan 12;5:1703356. doi: 10.3389/fbinf.2025.1703356 (PMC12832642; doi:10.3389/fbinf.2025.1703356)

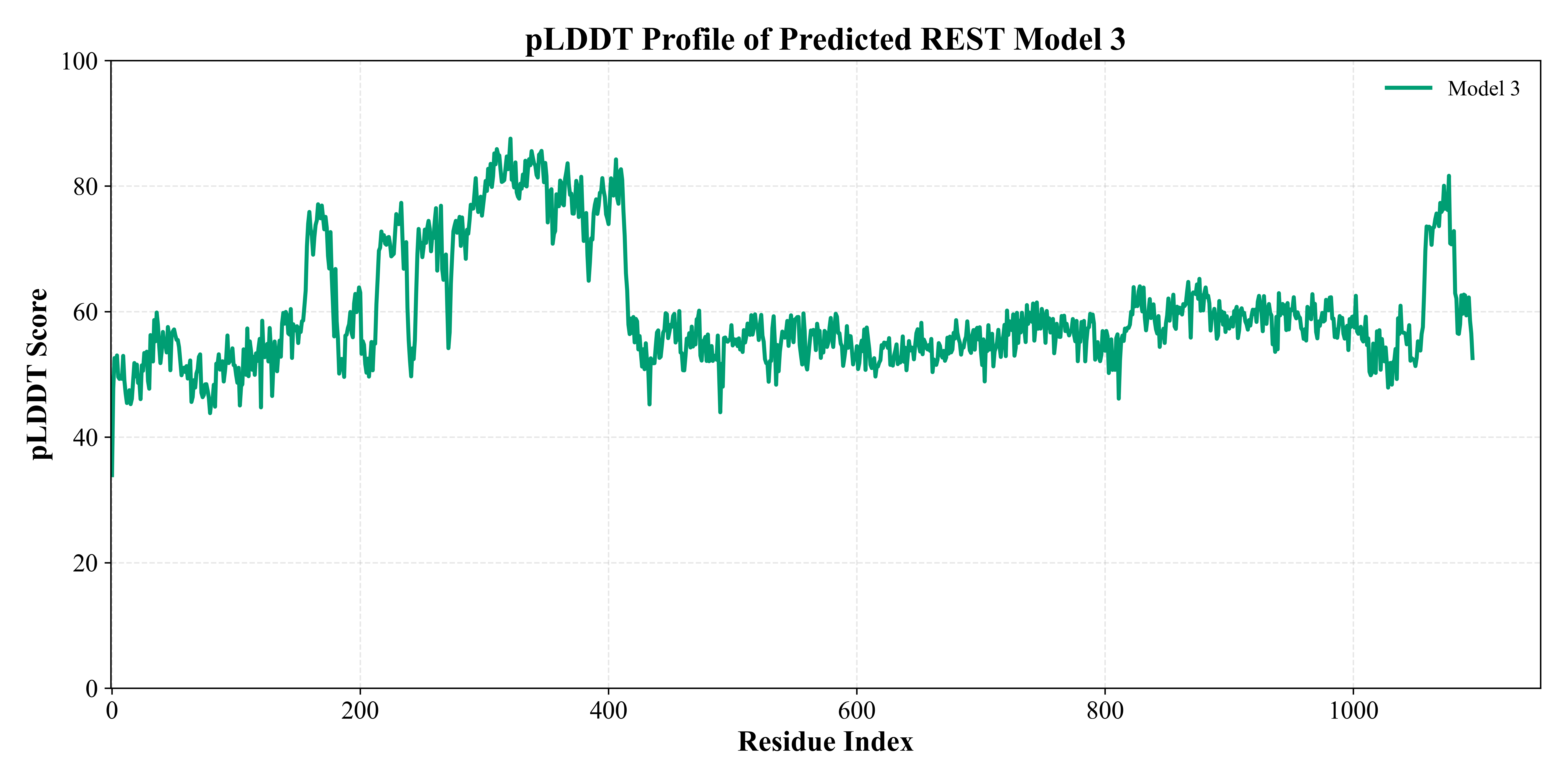

Supplement: Supplementary file 1 [file Image3.tiff]

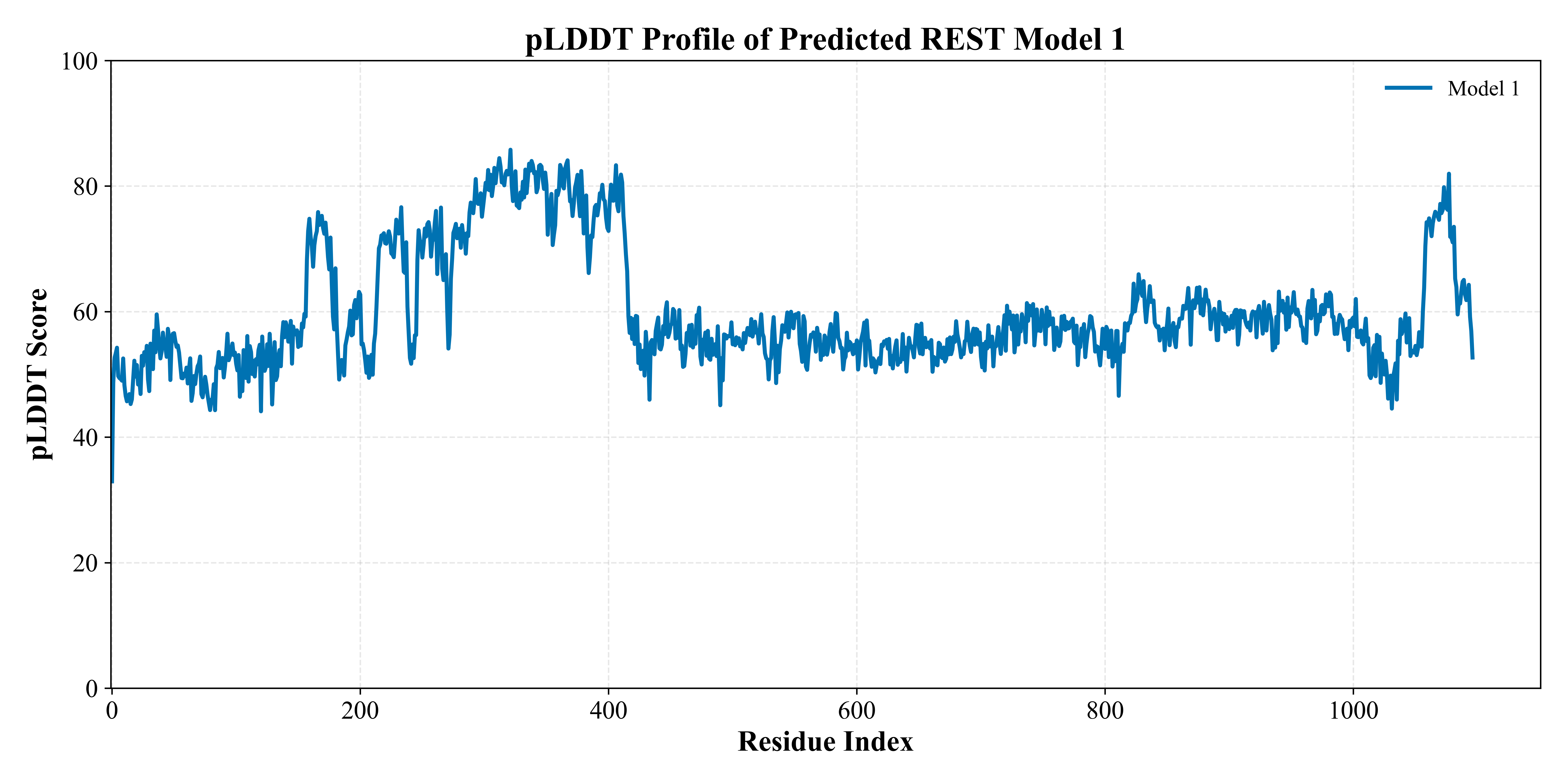

Supplement: Supplementary file 3 [file Image1.tiff]

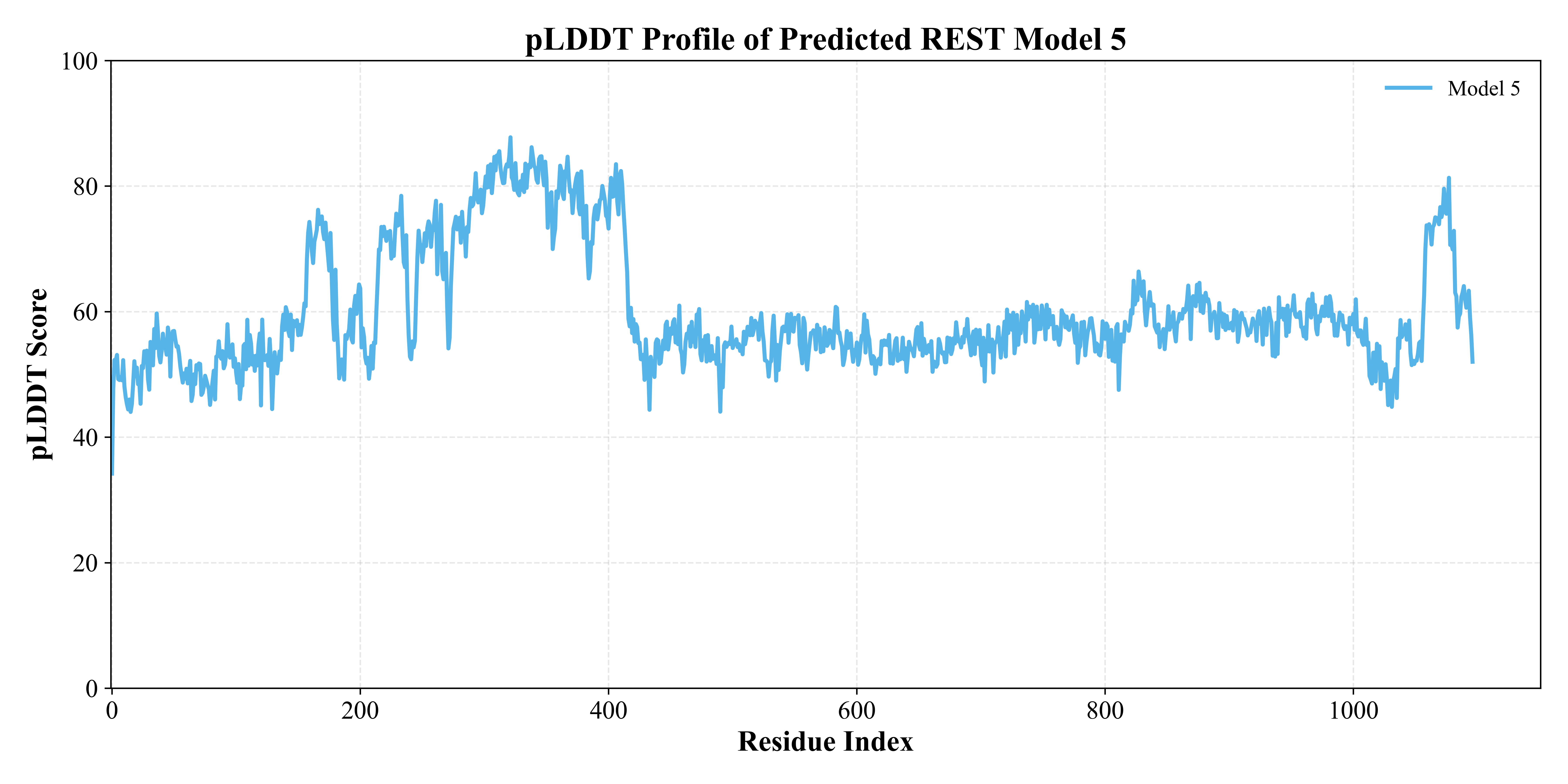

Supplement: Supplementary file 6 [file Image5.tiff]

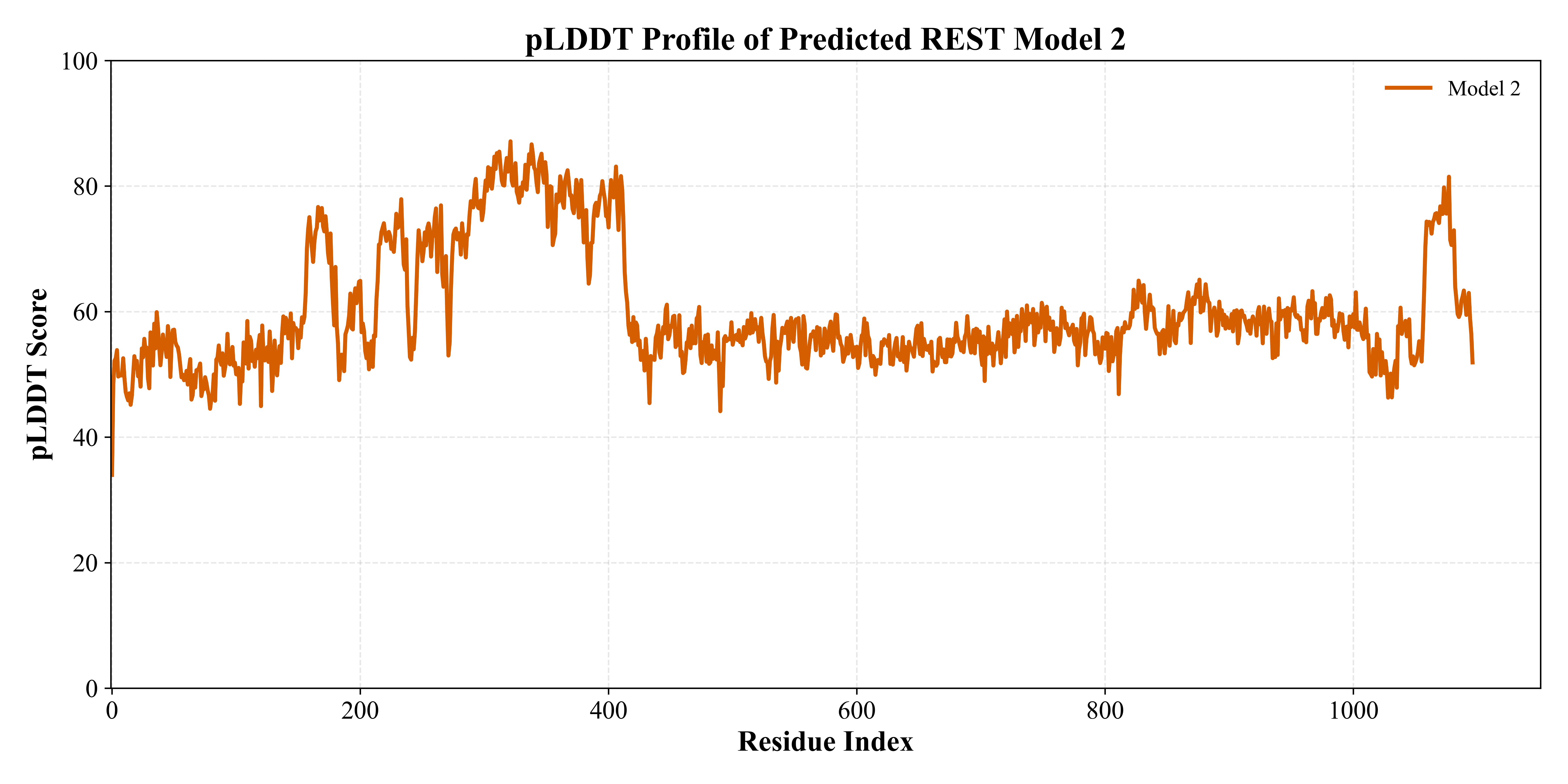

Supplement: Supplementary file 10 [file Image2.tiff]

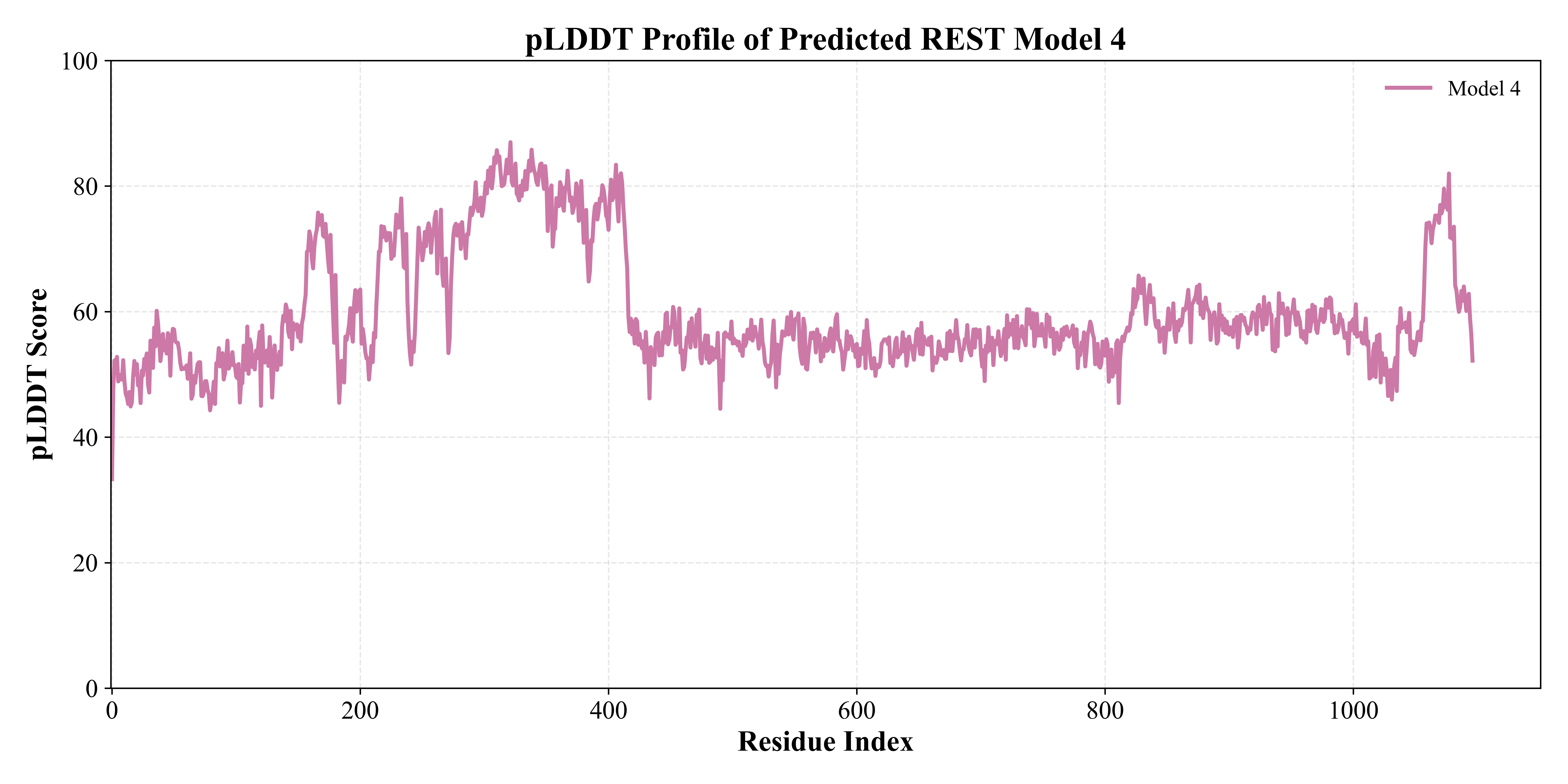

Supplement: Supplementary file 11 [file Image4.tiff]
