# Supplementary material for: REST missense mutations reveal disrupted Re1 motif binding and co-repressor interactions in uterine fibroids
Source: Front Bioinform. 2026 Jan 12;5:1703356. doi: 10.3389/fbinf.2025.1703356 (PMC12832642; doi:10.3389/fbinf.2025.1703356)
Supplement: Supplementary file 5 [file Table1.docx]

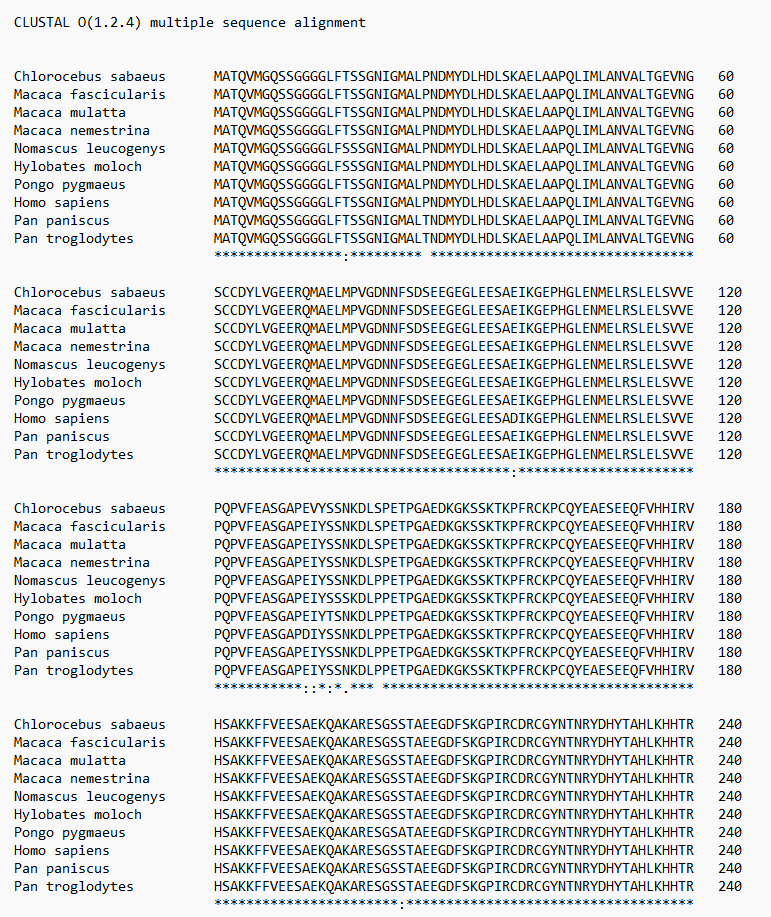


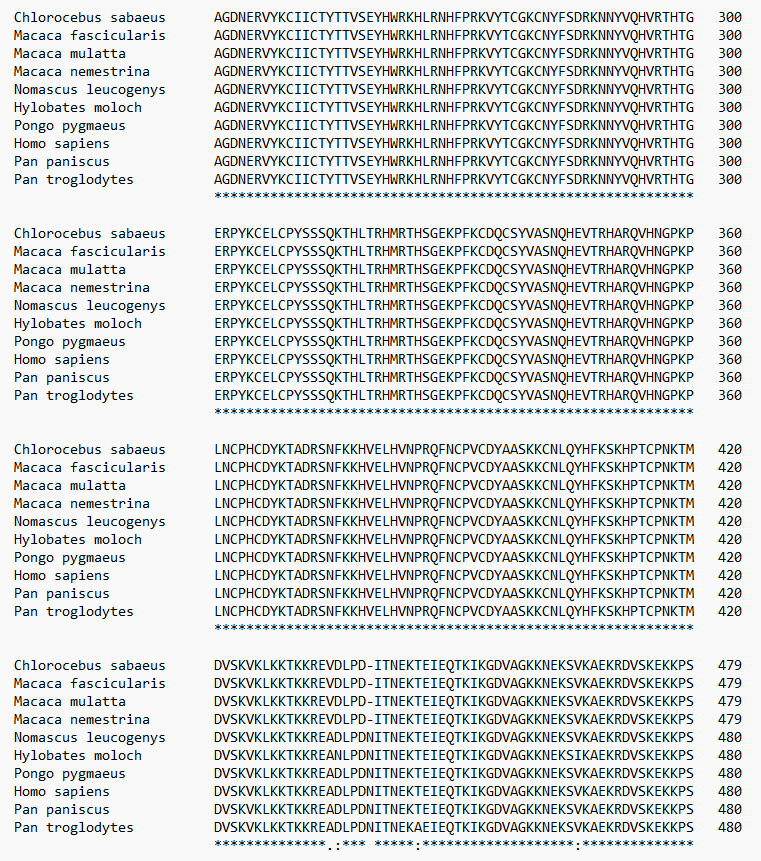


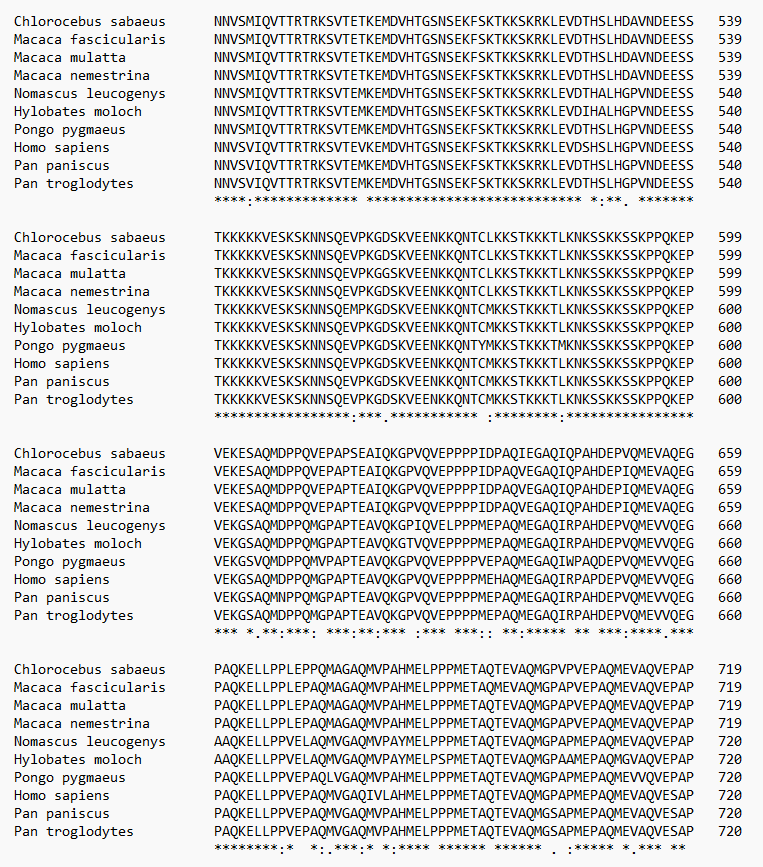


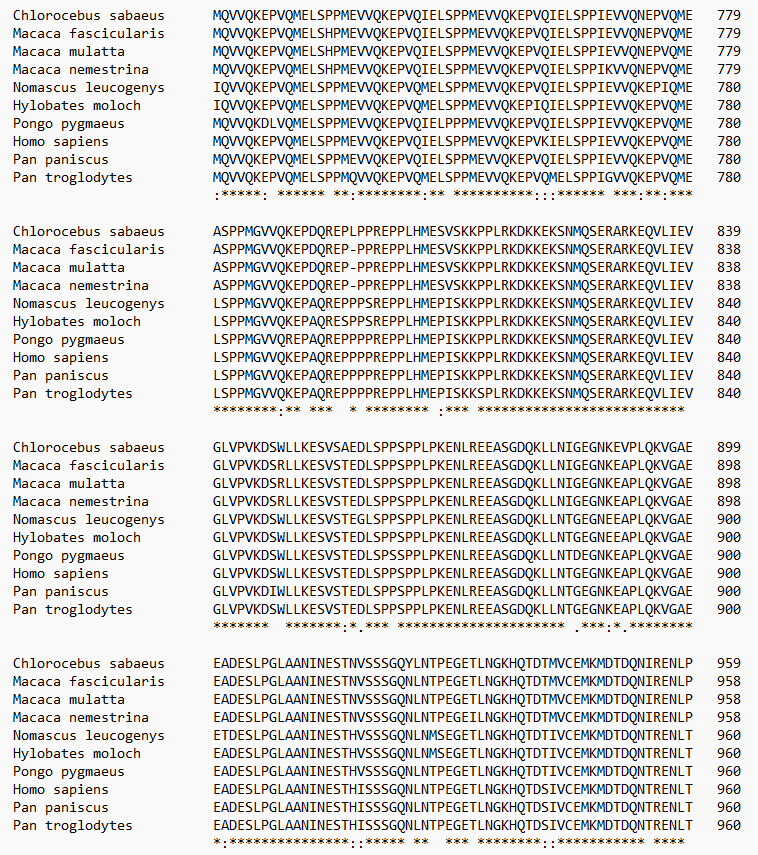


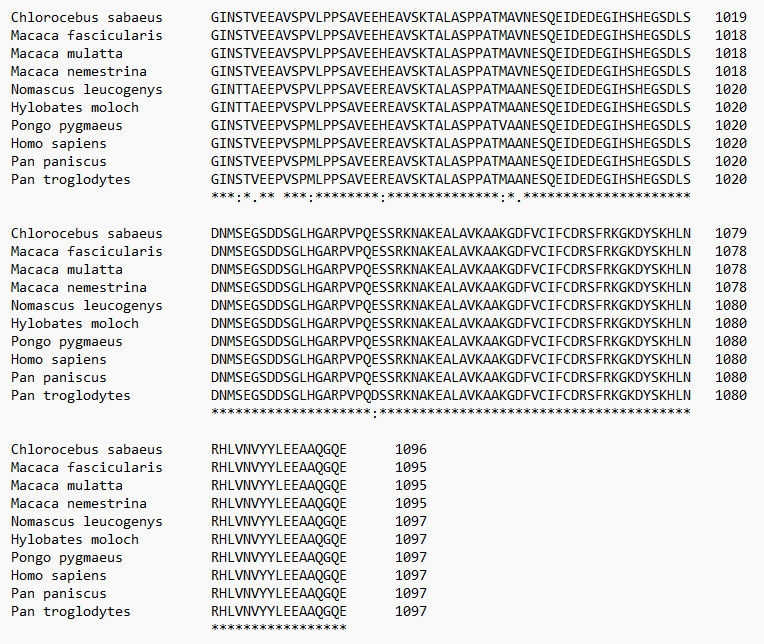


**Supplementary Fig. 1. Multiple sequence alignment (MSA) of REST protein across ten primate species using Clustal Omega. Conserved regions are indicated by asterisks (*), with functionally relevant residues affected by missense mutations (Y31, L76, Y283, L427) shown to be highly conserved across species.**

| Interactors | Haddock Score | Z-score | Cluster Size | RMSD | Van der Waals energy | Desolvation energy | Electrostatic energy | Buried Surface Area | Restraints violation energy |
| --- | --- | --- | --- | --- | --- | --- | --- | --- | --- |
| HDAC1 | -69.7 +/- 1.3 | -1.5 | 6 | 26.4 +/- 0.0 | -17.1 +/- 0.8 | 20.3 +/- 1.1 | -364.2 +/- 10.1 | 1223.8 +/- 113.5 | 0.0 +/- 0.0 |
| HDAC2 | -62.8 +/- 9.2 | -2.1 | 8 | 29.6 +/- 0.4 | -19.8 +/- 9.8 | 7.6 +/- 2.7 | -254.6 +/- 36.6 | 1150.2 +/- 124.7 | 2.7 +/- 2.5 |
| KDM1A | -87.5 +/- 7.4 | -2.2 | 6 | 0.9 +/- 0.7 | -19.3 +/- 5.2 | 31.9 +/- 5.5 | -500.9 +/- 60.6 | 1678.4 +/- 57.2 | 1.2 +/- 1.4 |
| RBBP7 | -82.6 +/- 0.5 | -1.5 | 117 | 6.0 +/- 0.2 | -22.5 +/- 6.1 | 11.9 +/- 3.6 | -360.1 +/- 46.1 | 1043.7 +/- 8.4 | 0.0 +/- 0.0 |
| SIN3A | -55.5 +/- 6.2 | -2.4 | 5 | 38.0 +/- 0.3 | -11.9 +/- 4.7 | 4.4 +/- 2.6 | -240.0 +/- 41.3 | 1056.6 +/- 57.5 | 0.3 +/- 0.6 |
| Re1 Motif | -22.8 +/- 3.1 | -2.0 | 12 | 6.2 +/- 0.4 | -26.2 +/- 2.2 | 15.4 +/- 1.8 | -171.9 +/- 21.6 | 1014.0 +/- 165.4 | 224.5 +/- 28.9 |

**Supplementary Table 1. HADDOCK docking statistics for wild-type REST with key co-repressors (HDAC1, HDAC2, KDM1A, RBBP7, SIN3A) and the Re1 motif.**

| Interactors | Haddock Score | Z-score | Cluster Size | RMSD | Van der Waals energy | Desolvation energy | Electrostatic energy | Buried Surface Area | Restraints violation energy |
| --- | --- | --- | --- | --- | --- | --- | --- | --- | --- |
| HDAC1 | -77.9 +/- 2.4 | -1.9 | 9 | 18.3 +/- 0.3 | -18.1 +/- 7.5 | 22.0 +/- 3.5 | -408.7 +/- 44.8 | 1365.9 +/- 110.3 | 0.0 +/- 0.0 |
| HDAC2 | -55.2 +/- 11.3 | -1.8 | 5 | 20.1 +/- 0.3 | 3.6 +/- 4.4 | 20.4 +/- 1.6 | -395.8 +/- 54.8 | 979.0 +/- 203.1 | 0.0 +/- 0.0 |
| KDM1A | -76.5 +/- 2.8 | -1.6 | 9 | 39.0 +/- 0.2 | -25.7 +/- 4.4 | 25.6 +/- 5.5 | -382.5 +/- 50.3 | 1434.3 +/- 22.5 | 0.4 +/- 0.5 |
| RBBP7 | -85.3 +/- 1.4 | -1.9 | 138 | 6.5 +/- 0.1 | -25.5 +/- 4.5 | 10.7 +/- 1.8 | -352.8 +/- 25.9 | 1065.2 +/- 49.5 | 0.1 +/- 0.1 |
| SIN3A | -65.5 +/- 2.3 | -1.6 | 11 | 33.3 +/- 0.5 | -2.8 +/- 13.1 | 25.1 +/- 1.8 | -441.6 +/- 79.3 | 1021.5 +/- 133.8 | 5.8 +/- 10.0 |
| Re1 Motif | -20.3 +/- 13.6 | -1.3 | 4 | 0.6 +/- 0.4 | -23.4 +/- 4.0 | 11.1 +/- 4.5 | -198.8 +/- 52.5 | 862.7 +/- 201.8 | 317.4 +/- 59.2 |

**Supplementary Table 2. Docking statistics for REST Mutant 1 (Y31C) with key corepressors (HDAC1, HDAC2, KDM1A, RBBP7, SIN3A) and the Re1 DNA motif.**

| Interactors | Haddock Score | Z-score | Cluster Size | RMSD | Van der Waals energy | Desolvation energy | Electrostatic energy | Buried Surface Area | Restraints violation energy |
| --- | --- | --- | --- | --- | --- | --- | --- | --- | --- |
| HDAC1 | -80.1 +/- 19.1 | -2.4 | 4 | 0.9 +/- 0.6 | -27.6 +/- 15.2 | 20.2 +/- 2.9 | -363.6 +/- 28.8 | 1483.8 +/- 283.6 | 0.0 +/- 0.0 |
| HDAC2 | -74.4 +/- 10.6 | -1.9 | 6 | 24.9 +/- 0.5 | -19.5 +/- 5.9 | 18.7 +/- 1.7 | -367.8 +/- 50.0 | 1379.2 +/- 122.1 | 0.0 +/- 0.0 |
| KDM1A | -84.9 +/- 3.9 | -1.9 | 10 | 15.6 +/- 0.1 | -24.0 +/- 6.1 | 29.9 +/- 1.5 | -454.0 +/- 35.9 | 1516.3 +/- 97.4 | 0.1 +/- 0.2 |
| RBBP7 | -85.7 +/- 1.6 | -2.2 | 124 | 0.7 +/- 0.4 | -25.4 +/- 4.3 | 12.9 +/- 2.7 | -365.8 +/- 35.0 | 1062.6 +/- 29.2 | 0.1 +/- 0.1 |
| SIN3A | -68.1 +/- 4.9 | -2.1 | 14 | 41.6 +/- 0.1 | -18.5 +/- 5.1 | 5.1 +/- 1.5 | -273.6 +/- 12.6 | 1271.1 +/- 165.0 | 0.2 +/- 0.3 |
| Re1 Motif | -25.3 +/- 4.1 | -1.3 | 10 | 9.5 +/- 0.2 | -26.3 +/- 8.0 | 11.6 +/- 4.6 | -145.2 +/- 22.6 | 864.2 +/- 118.2 |  |

**Supplementary Table 3. Docking statistics for REST Mutant 2 (Y31D) with key corepressors (HDAC1, HDAC2, KDM1A, RBBP7, SIN3A) and the Re1 DNA motif.**

| Interactors | Haddock Score | Z-score | Cluster Size | RMSD | Van der Waals energy | Desolvation energy | Electrostatic energy | Buried Surface Area | Restraints violation energy |
| --- | --- | --- | --- | --- | --- | --- | --- | --- | --- |
| HDAC1 | -73.6 +/- 12.6 | -2.1 | 4 | 0.8 +/- 0.5 | -19.8 +/- 0.9 | 19.2 +/- 2.3 | -365.0 +/- 76.1 | 1218.5 +/- 221.2 | 0.0 +/- 0.0 |
| HDAC2 | -61.8 +/- 17.6 | -1.7 | 4 | 22.2 +/- 0.1 | 8.0 +/- 2.2 | 16.9 +/- 3.7 | -433.1 +/- 101.7 | 835.6 +/- 252.9 | 0.0 +/- 0.0 |
| KDM1A | -66.4 +/- 7.9 | -1.5 | 20 | 11.1 +/- 1.3 | -22.4 +/- 7.2 | 19.5 +/- 2.4 | -318.0 +/- 36.5 | 1238.6 +/- 215.1 | 0.5 +/- 0.9 |
| RBBP7 | -86.9 +/- 4.0 | -1.9 | 153 | 0.7 +/- 0.4 | -24.9 +/- 2.0 | 10.3 +/- 3.2 | -362.0 +/- 30.2 | 1028.5 +/- 15.4 | 0.1 +/- 0.1 |
| SIN3A | -64.4 +/- 7.8 | -1.6 | 5 | 10.9 +/- 0.5 | -25.2 +/- 10.2 | -5.2 +/- 3.8 | -173.3 +/- 55.3 | 1259.9 +/- 158.8 | 6.0 +/- 9.1 |
| Re1 Motif | -22.2 +/- 7.5 | -1.7 | 4 | 7.8 +/- 0.5 | -37.4 +/- 6.7 | 18.6 +/- 2.8 | -136.4 +/- 46.9 | 1008.4 +/- 148.0 |  |

**Supplementary Table 4. Docking statistics for REST Mutant 3 (L76Q) with key corepressors (HDAC1, HDAC2, KDM1A, RBBP7, SIN3A) and the Re1 DNA motif.**

| Interactors | Haddock Score | Z-score | Cluster Size | RMSD | Van der Waals energy | Desolvation energy | Electrostatic energy | Buried Surface Area | Restraints violation energy |
| --- | --- | --- | --- | --- | --- | --- | --- | --- | --- |
| HDAC1 | -69.1 +/- 11.3 | -1.8 | 5 | 15.5 +/- 0.2 | -9.5 +/- 4.2 | 20.6 +/- 2.2 | -400.9 +/- 46.5 | 1121.4 +/- 254.7 | 0.0 +/- 0.0 |
| HDAC2 | -69.9 +/- 5.6 | -1.6 | 10 | 1.1 +/- 0.9 | -5.4 +/- 6.1 | 14.6 +/- 3.0 | -401.1 +/- 26.8 | 1112.9 +/- 64.3 | 10.3 +/- 16.1 |
| KDM1A | -75.3 +/- 11.4 | -1.5 | 11 | 30.9 +/- 0.2 | -7.3 +/- 5.8 | 28.3 +/- 3.9 | -481.9 +/- 64.7 | 1485.4 +/- 188.8 | 0.1 +/- 0.2 |
| RBBP7 | -90.3 +/- 2.6 | -2.0 | 116 | 0.6 +/- 0.4 | -27.9 +/- 3.7 | 11.2 +/- 2.2 | -367.8 +/- 17.0 | 1064.3 +/- 15.5 | 0.1 +/- 0.1 |
| SIN3A | -53.5 +/- 3.3 | -1.4 | 17 | 12.4 +/- 2.5 | -7.7 +/- 5.9 | 18.5 +/- 2.1 | -333.1 +/- 26.4 | 848.5 +/- 81.7 | 22.0 +/- 12.8 |
| Re1 Motif | -21.9 +/- 7.0 | -1.3 | 6 | 7.6 +/- 0.8 | -26.3 +/- 4.6 | 14.7 +/- 4.3 | -154.3 +/- 14.8 | 956.7 +/- 189.9 |  |

**Supplementary Table 5. Docking statistics for REST Mutant 4 (Y283C) with key corepressors (HDAC1, HDAC2, KDM1A, RBBP7, SIN3A) and the Re1 DNA motif.**

| Interactors | Haddock Score | Z-score | Cluster Size | RMSD | Van der Waals energy | Desolvation energy | Electrostatic energy | Buried Surface Area | Restraints violation energy |
| --- | --- | --- | --- | --- | --- | --- | --- | --- | --- |
| HDAC1 | -70.6 +/- 10.9 | -2.0 | 6 | 25.3 +/- 0.1 | -12.1 +/- 1.8 | 21.7 +/- 3.3 | -422.7 +/- 69.4 | 1046.8 +/- 98.5 | 44.4 +/- 0.7 |
| HDAC2 | -62.1 +/- 6.8 | -1.7 | 9 | 15.5 +/- 0.2 | -17.0 +/- 8.9 | 5.7 +/- 1.1 | -255.4 +/- 18.4 | 1124.5 +/- 180.2 | 2.7 +/- 1.9 |
| KDM1A | -73.0 +/- 10.0 | -1.4 | 4 | 36.6 +/- 0.1 | -13.7 +/- 8.9 | 24.9 +/- 4.2 | -423.4 +/- 80.4 | 1508.4 +/- 212.8 | 4.2 +/- 1.7 |
| RBBP7 | -86.3 +/- 2.0 | -2.0 | 141 | 0.5 +/- 0.3 | -31.5 +/- 1.8 | 11.4 +/- 0.7 | -331.2 +/- 7.0 | 1078.1 +/- 28.6 | 0.0 +/- 0.0 |
| SIN3A | -55.9 +/- 6.8 | -1.4 | 6 | 28.6 +/- 0.1 | -16.7 +/- 3.4 | 5.5 +/- 2.7 | -225.7 +/- 25.1 | 1118.7 +/- 49.8 | 5.2 +/- 8.9 |
| Re1 Motif | -22.7 +/- 10.5 | -1.7 | 10 | 11.2 +/- 0.5 | -21.8 +/- 8.2 | 15.6 +/- 2.1 | -177.2 +/- 5.2 | 820.8 +/- 193.5 |  |

**Supplementary Table 6. Docking statistics for REST Mutant 5 (L437Q) with key corepressors (HDAC1, HDAC2, KDM1A, RBBP7, SIN3A) and the Re1 DNA motif.**
